# Supplementary material for: PARP1 and PARP2 stabilise replication forks at base excision repair intermediates through Fbh1-dependent Rad51 regulation
Source: Nat Commun. 2018 Feb 21;9:746. doi: 10.1038/s41467-018-03159-2 (PMC5821833; doi:10.1038/s41467-018-03159-2)
Supplement: Supplementary file 1 — Supplementary Information [file 41467_2018_3159_MOESM1_ESM.pdf]

a

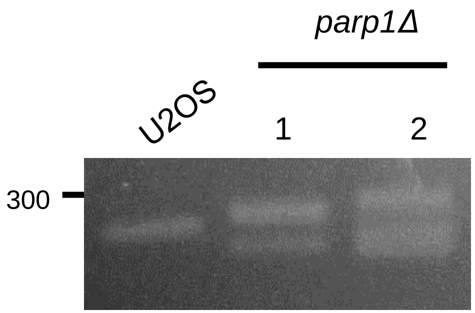

b

U2OS

ACTTTCTGCTGGTCATCCCACCGAAGCTCAGAGAACCCATCCACCTCAACGTCAGGGTGCCGGATGGAGTGGCCCACCTTCCAG

parp1Δ 1

+5bp ACTTTCTGCTGGTCATCCCACCGAAGCGAAGCTCAGAGAACCCATCCACCTCAACGTCAGGGTGCCGGATGGAGTGGCCCACCTTCCAG

-23bp ACTTTCTGCTGGTCATC - CCACCTCAACGTCAGGGTGCCGGATGGAGTGGCCCACCTTCCAG

parp1Δ 2

+8bp ACTTTCTGCTGGTCATCCCACCGAAGCTCAGAAGCTCAGAGAACCCAT CCACCTCAACGTCAGGGTGCCGGATGGAGTGGCCCACCTTCCAG

-31bp ACTTTCTGCTGGTCATCCCACCGAAGCTCA - GGATGGAGTGGCCCACCTTCCAG

Supplementary Figure 1: Validation of *parp1Δ* cell lines by sequencing of indels
a. PCR across the CRISPR targeted locus within the *PARP1* gene and analysis of indels in *parp1Δ* cell lines by gel electrophoresis.
b. Sanger sequencing of indels identified in (A). Red bases indicate insertions, dashes indicate deletions, green bases indicate substitutions.

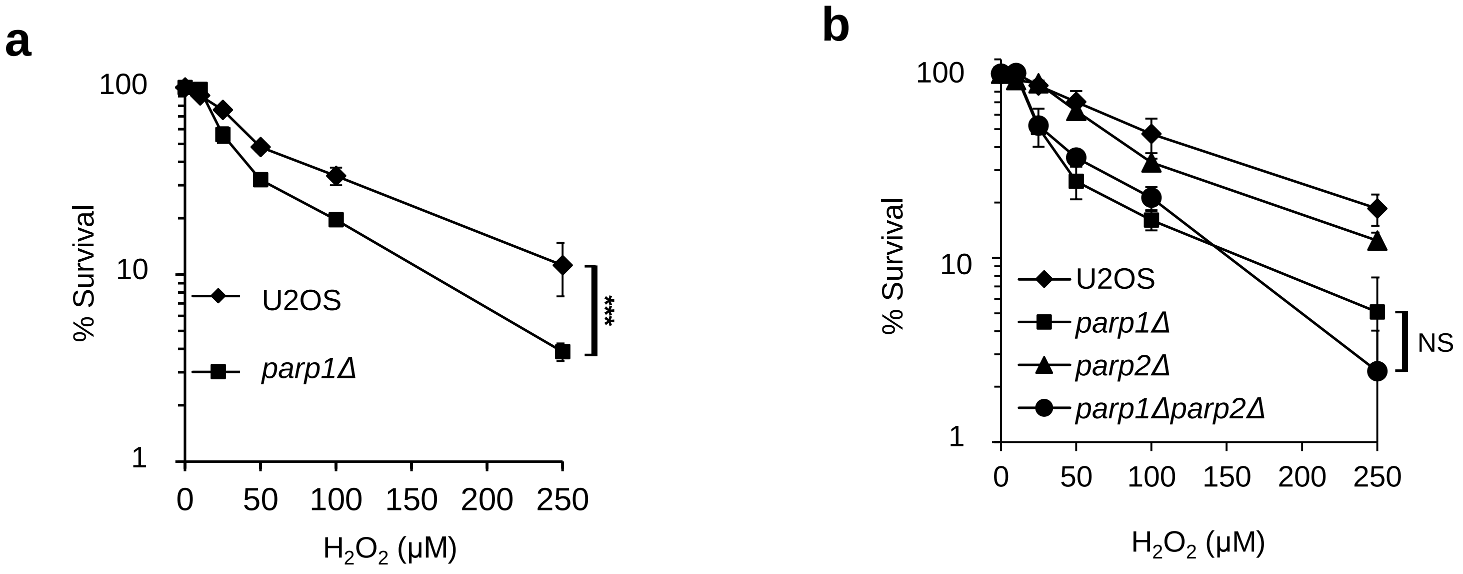

Supplementary Figure 2: Disruption of *PARP2* does not further sensitise *parp1Δ* cells to H_2O_2

a. U2OS or *parp1Δ* cells were exposed to the indicated concentrations of H_2O_2 and cell viability assessed by a clonogenic survival assay. Error bars represent the SEM from three independent experiments. Statistical significance was determined by two-way ANOVA (NS, not significant; *** p<0.001). **b.** U2OS, *parp1Δ, parp2Δ* and *parp1/2Δ* cells were exposed to the indicated concentrations of H_2O_2 and cell viability assessed by a clonogenic survival assay. Error bars represent the SEM from three independent experiments. Statistical significance was determined as in (A).

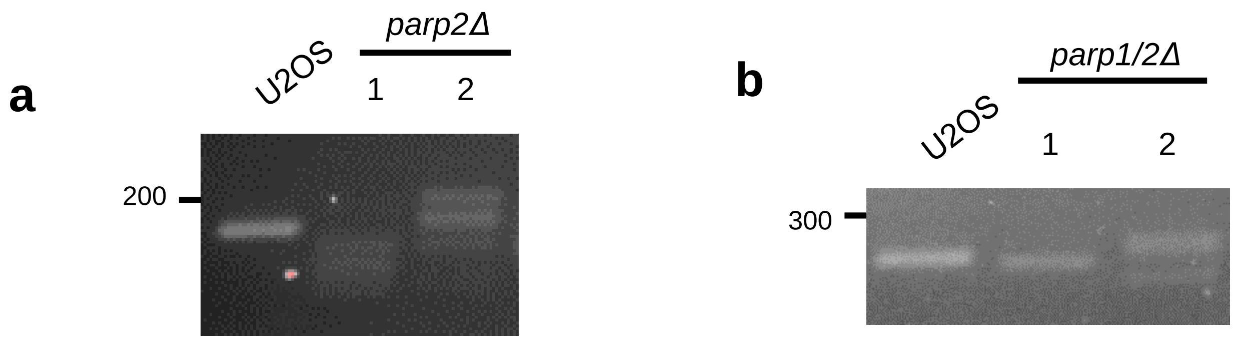

c

U2OS

aaagtagtacctatctgtctttcctcagAATCTGTGAAGGCCTTGCTGTTAAAGGGCAAAGCTCCTGTGGACCCAGAGTGTACAG

parp2Δ 1

-31bp aaagtagtacctatccgtctttcctcagAATCTGTGAAGG - CCCAGAGTGTACAG

-40bp aaagtagtacctatctgtctttcctcagAATCTGT - GAGTGTACAG

-46bp aaagtagtacctatctgtctt - TGGACCCAGAGTGTACAG

-50bp aaagtagtacctatctgtctttcctc - AGTGTACAG

parp2Δ 2

+8bp aaagtagtacctatctgtctttcctcagAATCTGTGAAGGCCTTGCTGTTAAAGGGCAAAGCTGCAAAGCTCCTGTGGACCCAGAGTGTACAG

+1bp aaagtagtacctatctgtctttcctcagAATCTGTGAAGGGCAAAGCTCGTGAAGGGCAAAGCTCCTGTGGACCCAGAGTGTACAG

-14bp aaagtagtacctatctgtctttcctcagAATCTGTG - - - - - - - - - - - - - - AAAGGGCAAAGCTCCTGTGGACCCAGAGTGTACAG

U2OS

ACTTTCTGCTGGTCATCCCACCGAAGCTCAGAGAACCCATCCACCTCAACGTCAGGGTGCCGGATGGAGTGGCCCACCTTCCAG

parp1/2Δ 1

-7bp ACTTTCTGCTGGTCATCCCACCGAAGGTCAGGG - - - - - - - CCACTCAACGTCAGGGTGCCGGATGGAGTGGCCCACCTTCCAG

parp1/2Δ 2

+4bp ACTTTCTGCTGGTCATCCCACCGAAGCTAGCTCAGAGAACCCATCCACCTCAACGTCAGGGTGCCGGATGGAGTGGCCCACCTTCCAG

-25bp ACTTTCTGCTGGTCATCCCA - TCAACGTCAGGGTGCCGGATGGAGTGGCCCACCTTCCAG

Supplementary Figure 3: Validation of *parp2Δ* and *parp1/2Δ* cell lines by sequencing of indels
a. PCR across the CRISPR targeted locus within the *PARP2* gene and analysis of indels in *parp2Δ* cell lines by gel electrophoresis. **b.** The *parp1/2Δ* cell lines were generated by disrupting the *PARP1* gene in *parp2Δ cells.* PCR across the CRISPR targeted locus within the *PARP1* gene and analysis of indels in *parp1/2Δ* cell lines by gel electrophoresis are illustrated. **c.** Sanger sequencing of these indels confirms they produce exonic frameshift mutations, or disrupt the splice site, and prevent expression of PARP1 or PARP2. Red bases indicate insertions, dashes indicate deletions, green bases indicate substitutions.

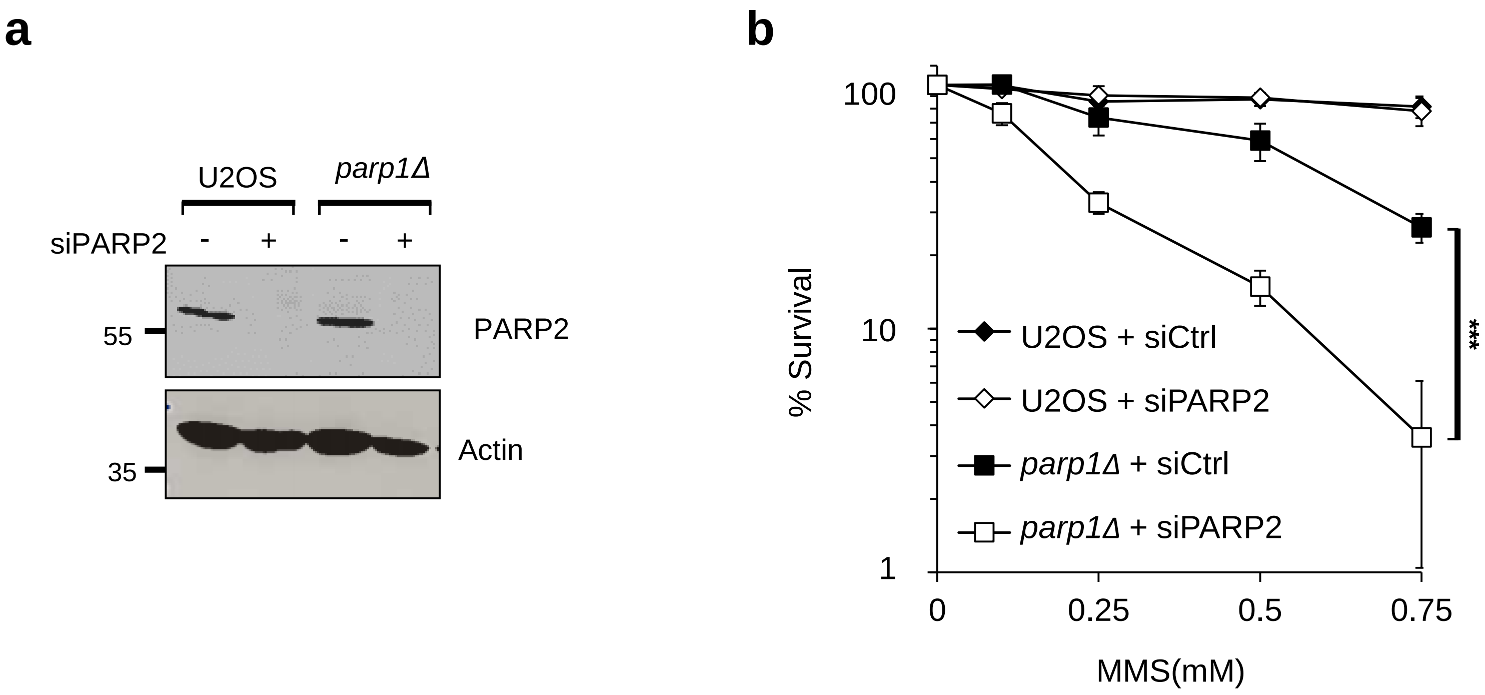

Supplementary Figure 4: Depletion of PARP2 sensitises *parp1Δ* cells to MMS
a. Whole cell extracts were prepared from U2OS or *parp1Δ* cells transfected with Ctrl or PARP2 siRNA and analysed by Western blotting using the indicated antibodies. **b.** U2OS or *parp1Δ* cells transfected with Ctrl or PARP2 siRNA were exposed to the indicated concentrations of MMS and cell viability assessed by a clonogenic survival assay. Error bars represent the SEM from three independent experiments. Statistical significance was determined by two-way ANOVA (*** $p<0.001$).

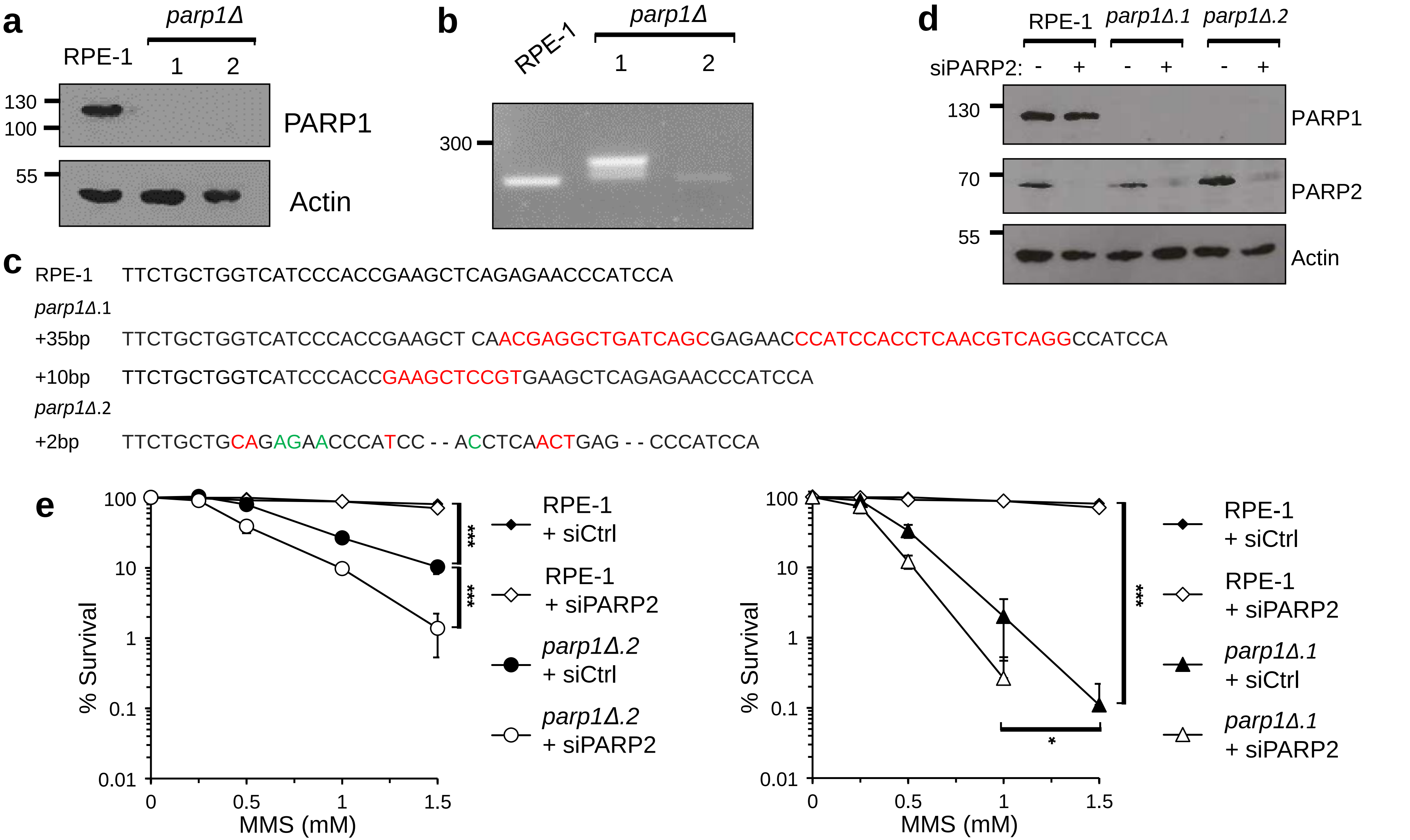

Supplementary Figure 5: Depletion of PARP2 sensitises *parp1Δ* RPE-1 cells to MMS

a. Whole cell extracts were prepared from RPE-1 or two *parp1Δ* RPE-1 cell lines and Western blotting performed with the indicated antibodies. **b.** PCR across the CRISPR targeted locus within the *PARP1* gene and analysis of indels in *parp1Δ* RPE-1 cell lines by gel electrophoresis. **c.** Sanger sequencing of indels identified in (B). Red bases indicate insertions, dashes indicate deletions, green bases indicate substitutions. **d.** Whole cell extracts were prepared from the indicated cell lines after transfection with siCtrl or siPARP2, and Western blotting performed with the indicated antibodies**. e.** RPE-1 or *parp1Δ* RPE-1 cell lines, transfected with siCtrl or siPARP2, were exposed to MMS and cell survival assessed by clonogenic assays. Error bars represent the SEM from three independent experiments. Statistical significance was determined by two-way ANOVA (* $p < 0.05$; *** $p<0.001$).

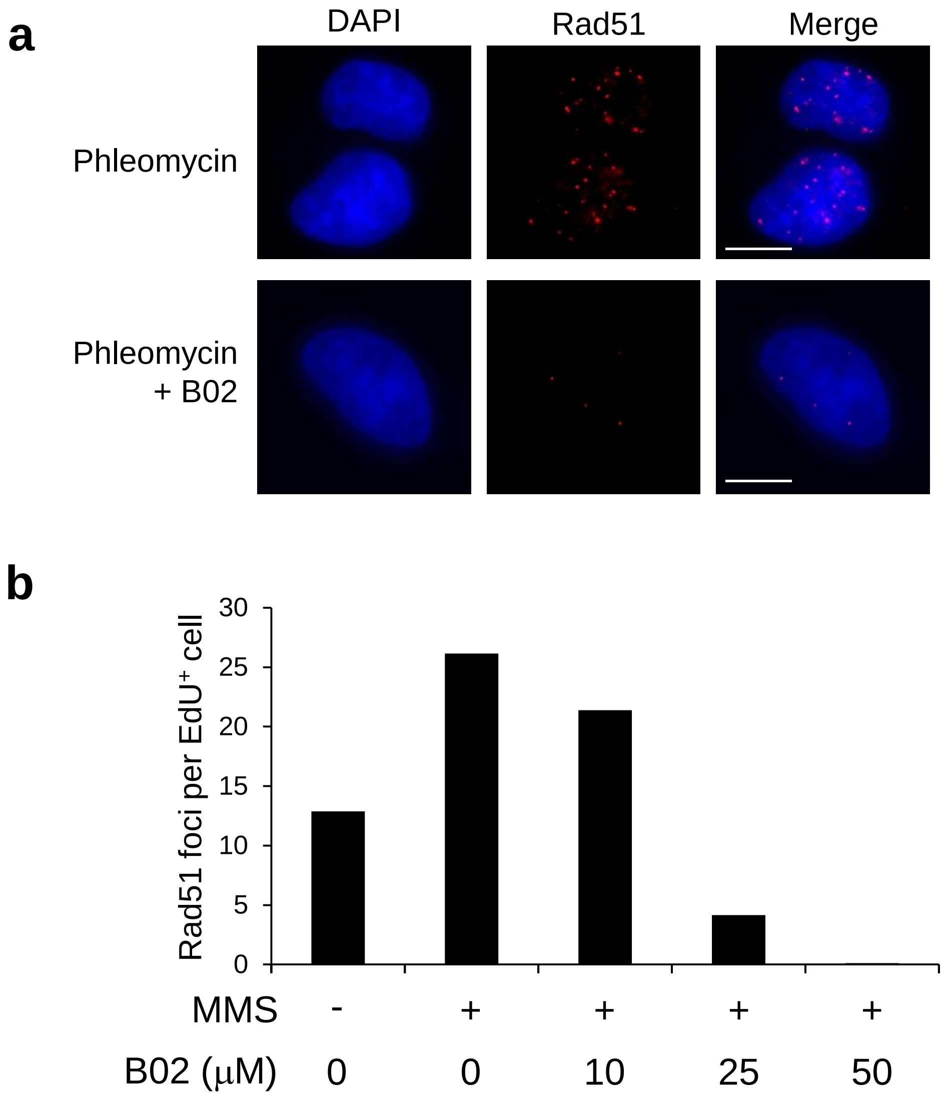

Supplementary Figure 6: B02 disrupts Rad51 foci in U2OS cells

a. U2OS cells were treated with carrier (DMSO) or 30 mM B02 for 1 hour prior to the addition of 300 mM phleomycin for 1 hour. Cells were then allowed to recover in fresh media containing B02 for 5 hours. Rad51 foci were detected by immunofluorescence. Scale bars represent 10 μm. **b.** U2OS cells were treated with carrier (DMSO) or the indicated concentrations of B02 for 1 hour prior to addition of 0.5 mM MMS and 1 μM EdU for 1 hour. Cells were then allowed to recover in fresh media containing B02 for 5 hours. Rad51 foci were detected by immunofluorescence and the number of Rad51 foci in EdU positive nuclei quantified.

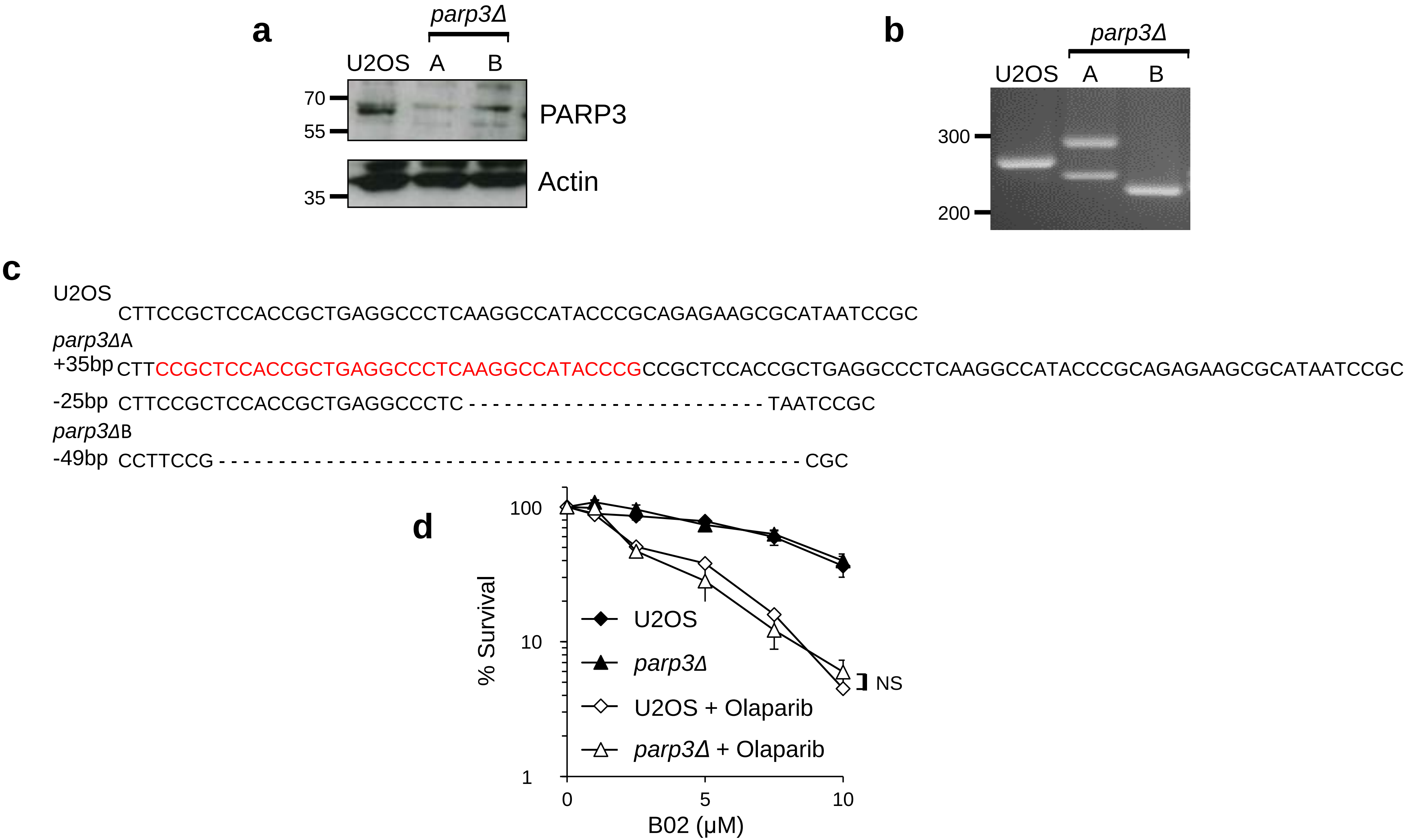

Supplementary Figure 7: Depletion of PARP3 is not synthetic lethal with HR inhibition

a. Nuclear extracts were prepared from U2OS or two *parp3Δ* cell lines and Western blotting performed with the indicated antibodies**.** **b.** PCR across the CRISPR targeted locus within the *PARP3* gene and analysis of indels in *parp3Δ* cell lines by gel electrophoresis. **c.** Sanger sequencing of indels identified in (A). Red bases indicate insertions, dashes indicate deletions, green bases indicate substitutions. **d.** U2OS or *parp3Δ* cells were exposed to Olaparib and B02 as indicated. Cell viability was determined using a clonogenic survival assay. Error bars represent the SEM from three independent experiments. Statistical significance was determined by two-way ANOVA (NS, not significant).

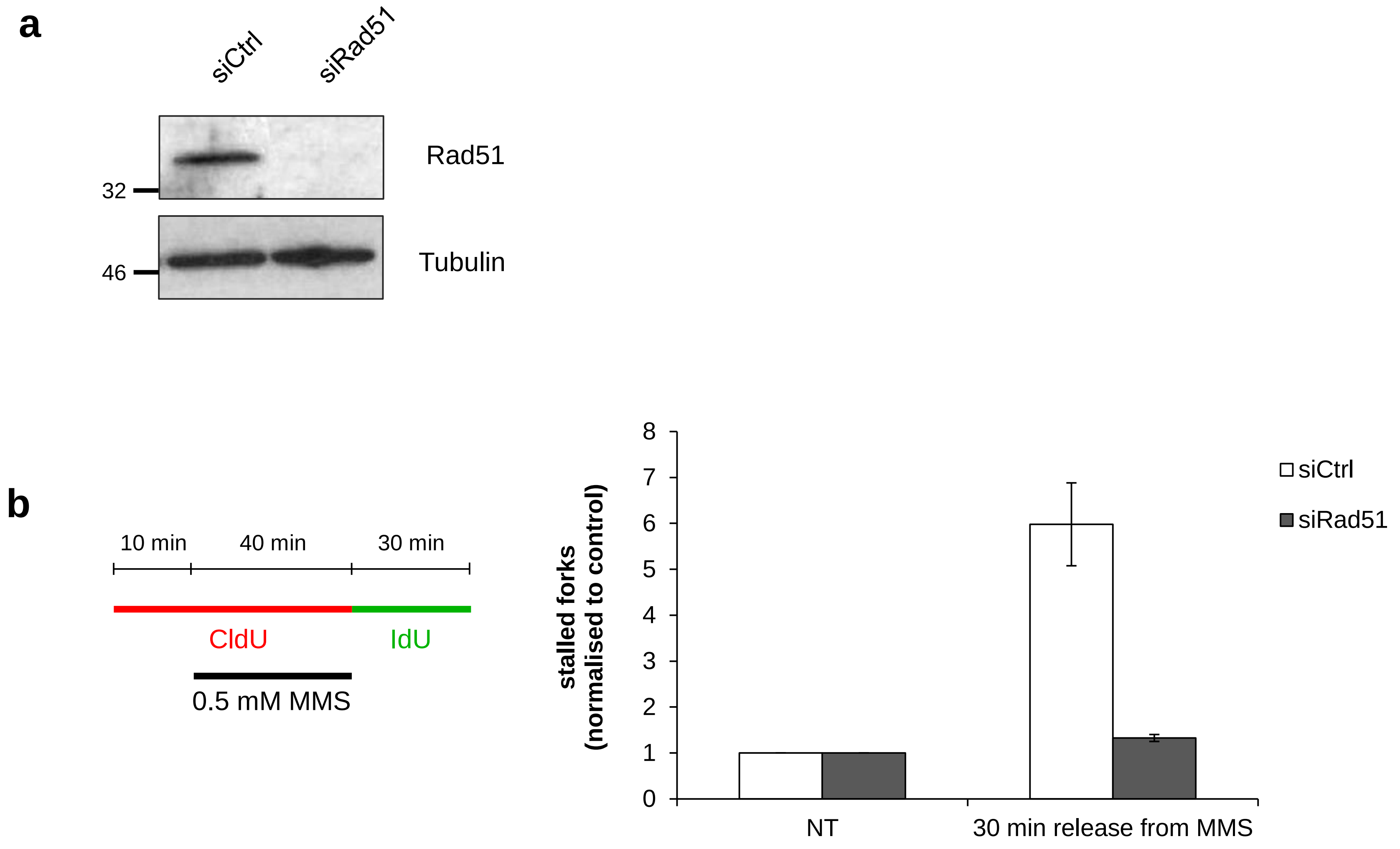

Supplementary Figure 8: Rad51 depletion suppresses fork stalling in response to MMS
a. Whole cell extracts were prepared from U2OS cells transfected with Ctrl or Rad51 siRNA and analysed by Western blotting using the indicated antibodies. **b.** DNA fibre analysis was carried out on U2OS transfected with Ctrl or Rad51 siRNA after MMS exposure and recovery for the indicated times. The number of stalled forks (red-only tracts) was determined as a ratio of all red-labelled replication structures and subsequently normalised to the ratio in the corresponding non-treated sample. Error bars represent the SEM from at least three independent experiments.

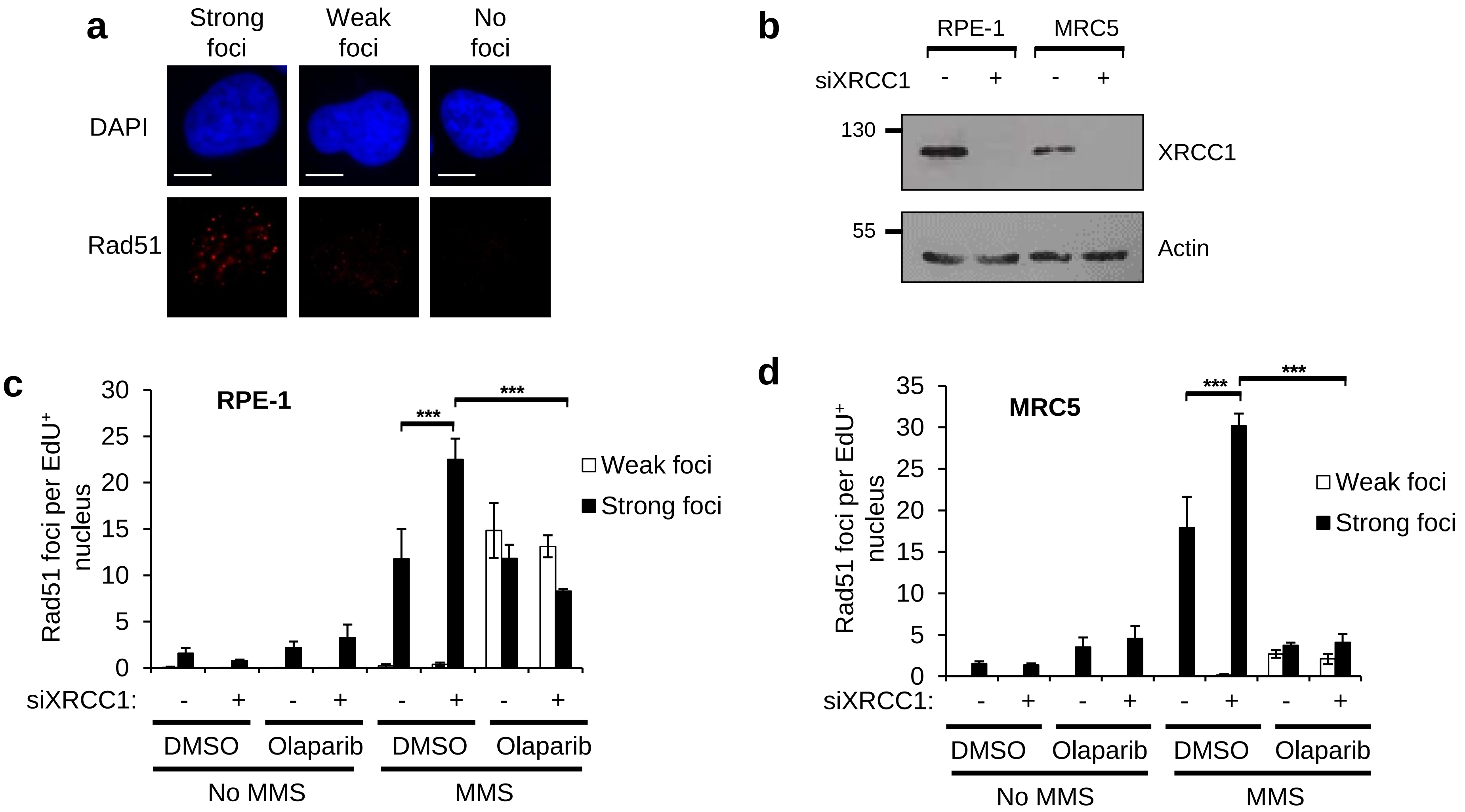

Supplementary Figure 9: PARPi compromise Rad51 foci formation after MMS exposure in multiple independent cell lines
a. RPE-1 cells were treated with 1 μM Olaparib for 1 hour, followed by 1 μM EdU and 0.5 mM MMS for 1 hour, before recovery in fresh media. Six hours after MMS addition, cells were fixed, and EdU and Rad51 were detected by immunofluorescence. Representative images of EdU-positive cells showing strong, weak or no Rad51 foci are shown. Scale bars represent 10 μm. **b.** Whole cell extracts were prepared from RPE-1 or MRC5 cells after transfection with siCtrl or siXRCC1 and Western blotting performed using the indicated antibodies. **c. and d.** The indicated cell lines, transfected with siCtrl or siXRCC1, were treated as in (A) and Rad51 nuclear foci analysed in EdU positive cells. Cells were fixed 12 hours after addition of EdU and MMS. Error bars represent the SEM from three independent experiments. Statistical significance was determined using a two-tailed Student's t-test (*** $p<0.001$).

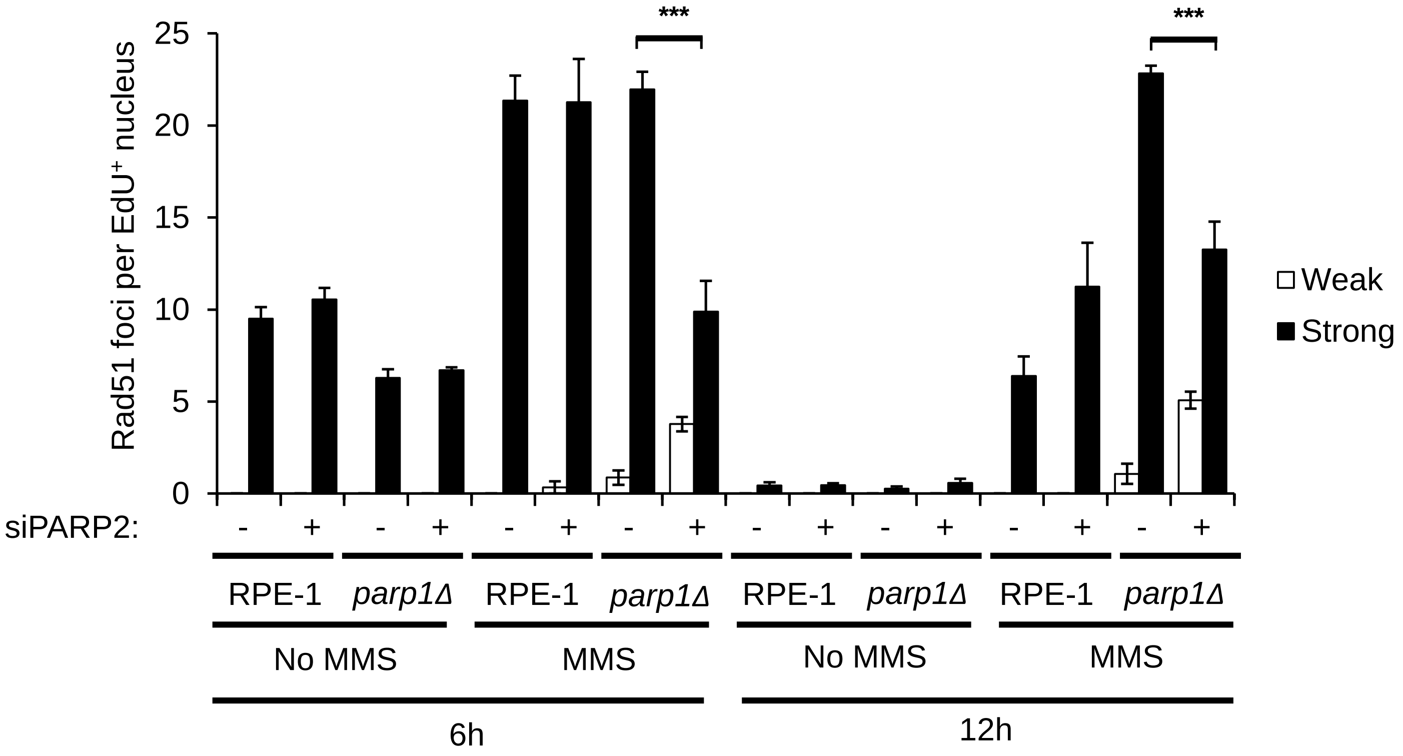

Supplementary Figure 10: RPE-1 cells lacking PARP1 and PARP2 show compromised Rad51 foci formation after MMS exposure.

The indicated cell lines, transfected with siCtrl or siPARP2, were treated with 1 μM EdU and 0.5 mM MMS for 1 hour, before recovery in fresh media. Cells were fixed at the indicated times after addition of EdU and MMS and Rad51 nuclear foci analysed in EdU positive cells. Error bars represent the SEM from three independent experiments. Statistical significance was determined using a two-tailed Student's t-test (*** $p<0.001$).

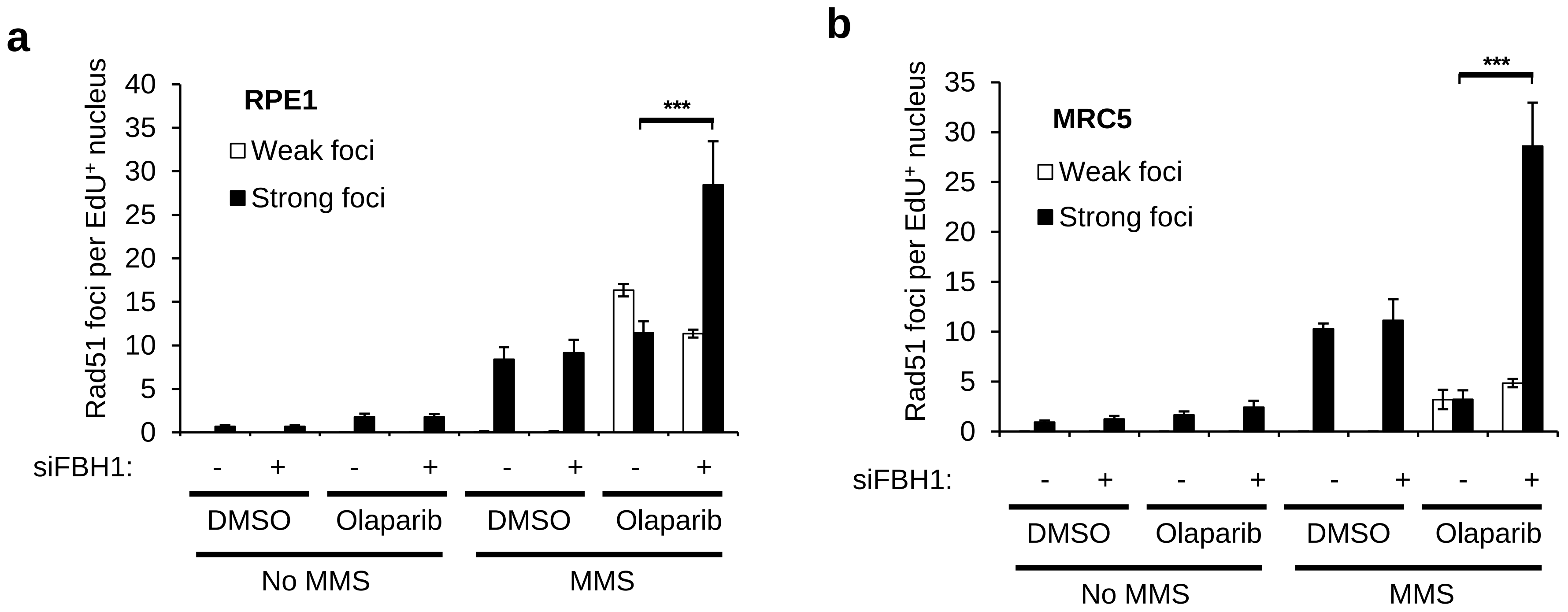

Supplementary Figure 11: Loss of Fbh1 can restore Rad51 foci formation after PARPi exposure in multiple independent cell lines

a. and b. The indicated cell lines, transfected with siCtrl or siFbh1, were treated with 1 μM Olaparib for 1 hour, followed by 1 μM EdU and 0.5 mM MMS for 1 hour, before recovery in fresh media. Cells were fixed 12 hours after addition of EdU and MMS and Rad51 nuclear foci analysed in EdU positive cells. Error bars represent the SEM from three independent experiments. Statistical significance was determined using a two-tailed Student's t-test (*** $p<0.001$).

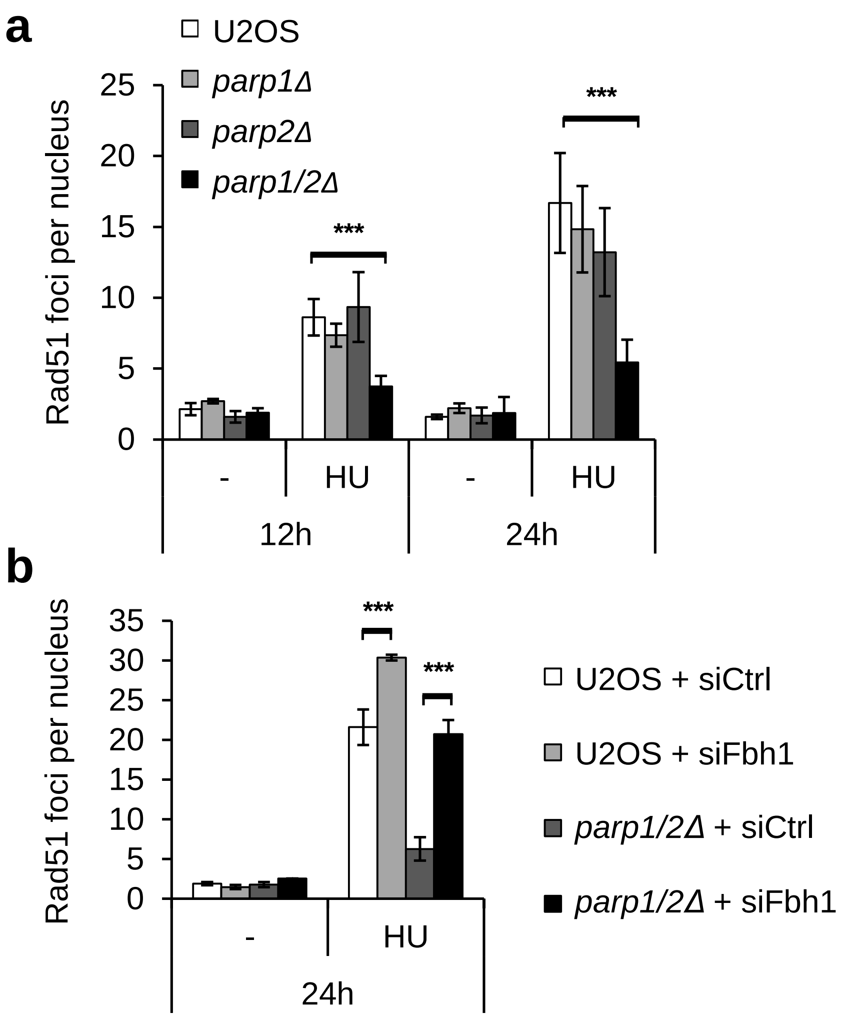

c

	Average IdU:CldU ratio	SEM	p value
U2OS siRNA + HU	0.99	0.01	-
parp1Δ + HU	0.70	0.02	<0.001
parp2Δ + HU	0.86	0.02	<0.001
parp1/2Δ + HU	0.60	0.01	<0.001

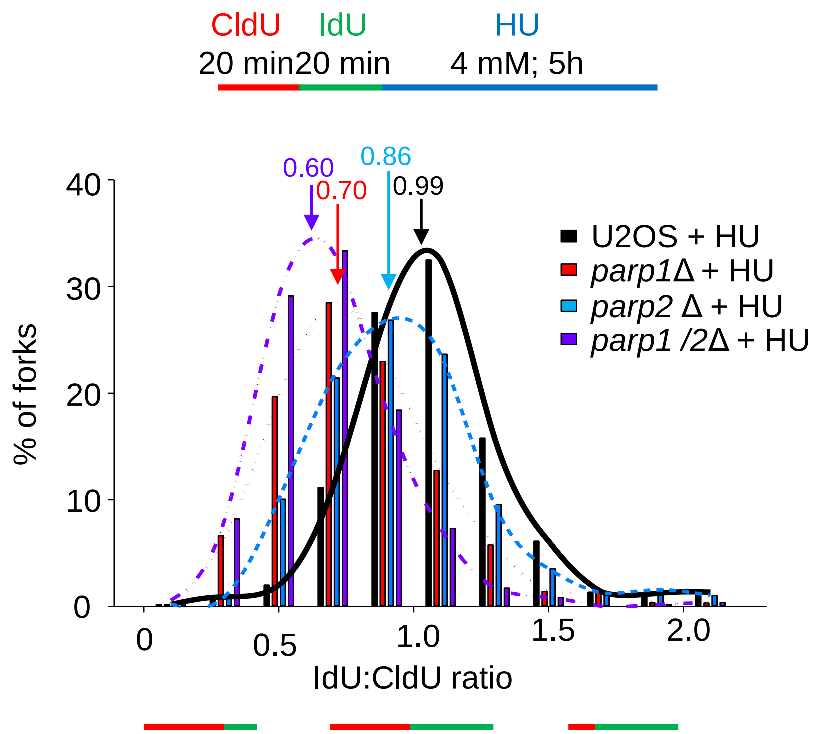

Supplementary Figure 12: Rad51 foci and replication fork stability is compromised after HU exposure in the absence of PARP1 and PARP2

a. The indicated cell lines were treated with 2 mM HU for the indicated times, and Rad51 nuclear foci analysed by immunofluorescence. Error bars represent the SEM from three independent experiments. Statistical significance was determined using a two-tailed Student's t-test (*** $p<0.001$). **b.** The indicated cell lines, transfected with siCtrl or siFbh1, were treated with 2 mM HU for the 24 hours, and Rad51 nuclear foci analysed by immunofluorescence. Error bars represent the SEM from three independent experiments. Statistical significance was determined as in (A). **c.** Fork degradation was analysed in the indicated cell lines. Cells were pulsed for 20 min each with CldU and IdU, then exposed to HU for 5 h. DNA was visualised with antibodies against CldU and IdU, and the plot display the average ratios of IdU:CldU label lengths from three independent experiments. The arrows indicate mean values, and statistical differences were analysed with a Mann-Whitney rank sum test.

Figure 1

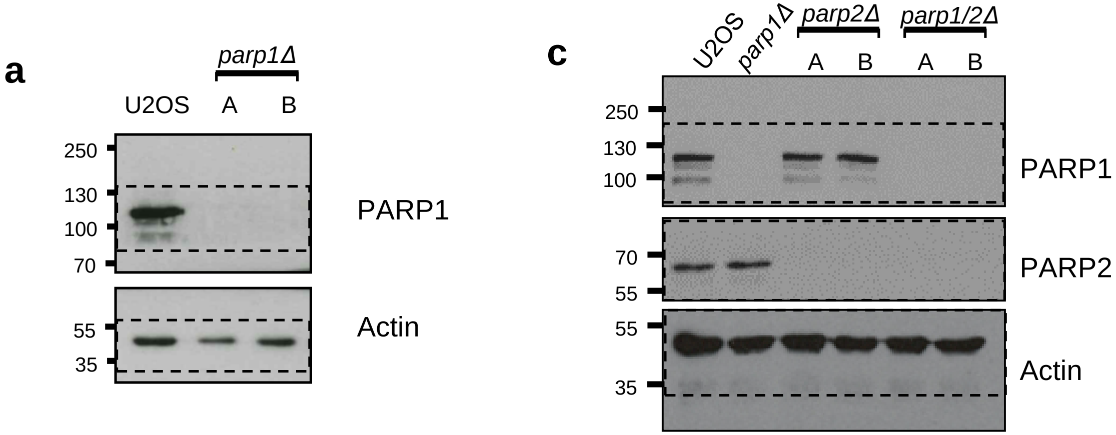

Figure 3

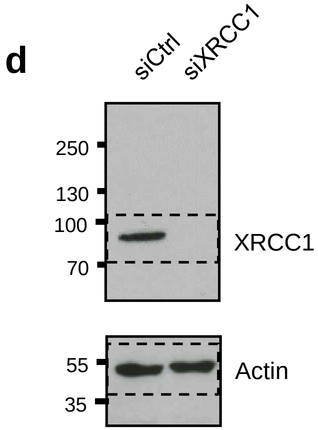

Supplementary Figure 13: Uncropped images of blots and gels

Figure 5

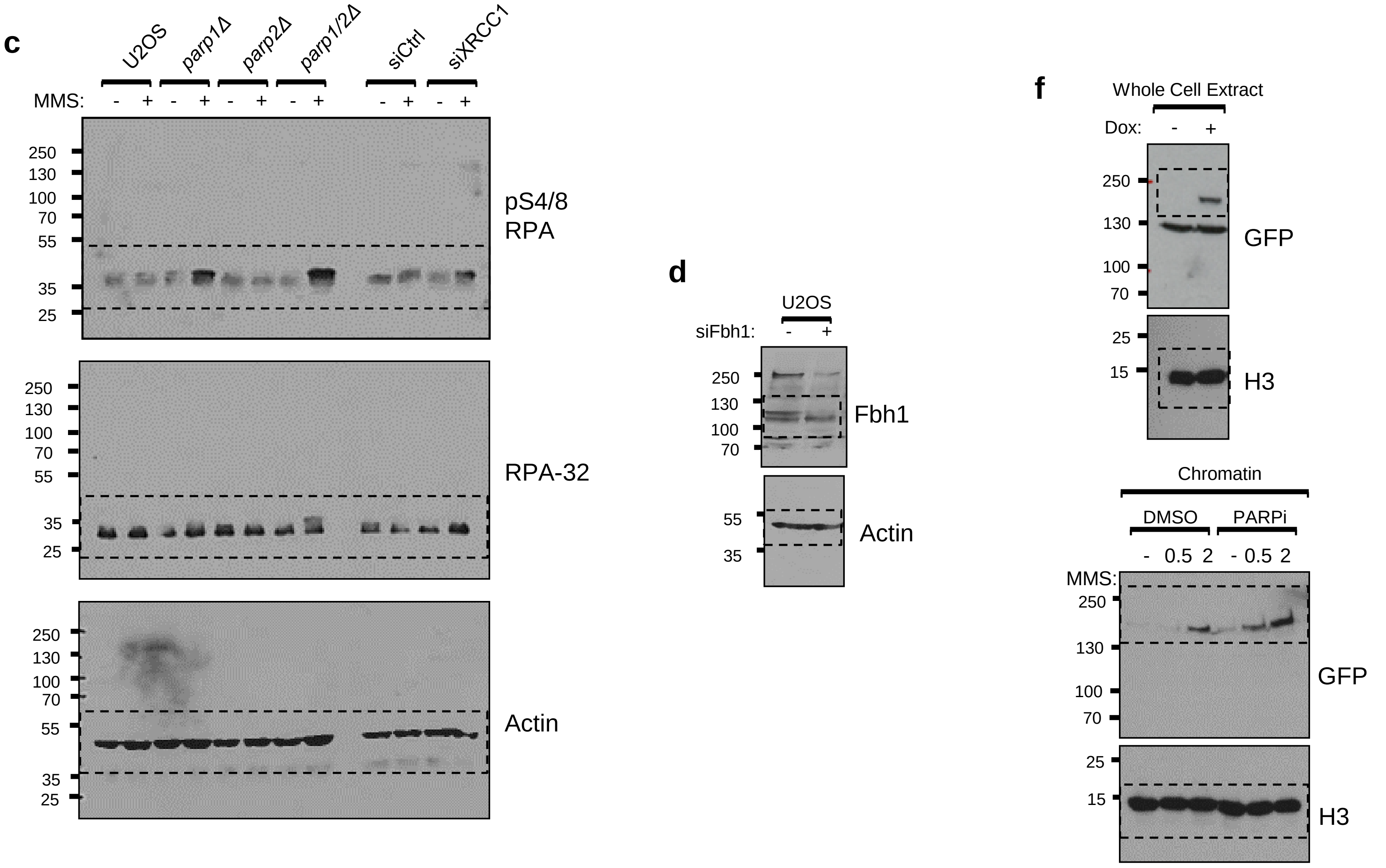

Supplementary Figure 13: Uncropped images of blots and gels

Supplementary Figure 1

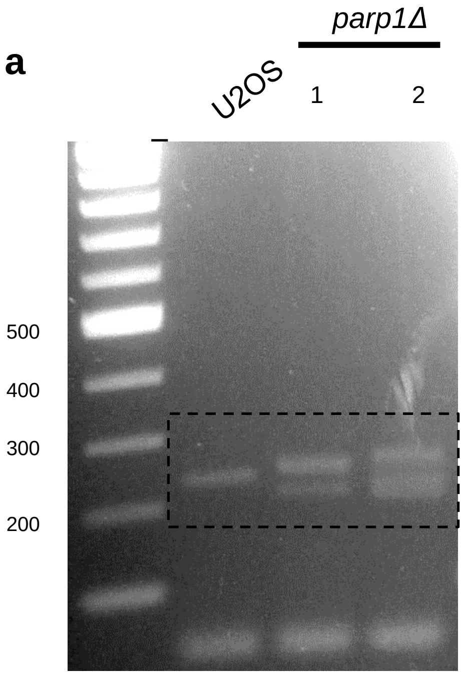

Supplementary Figure 3

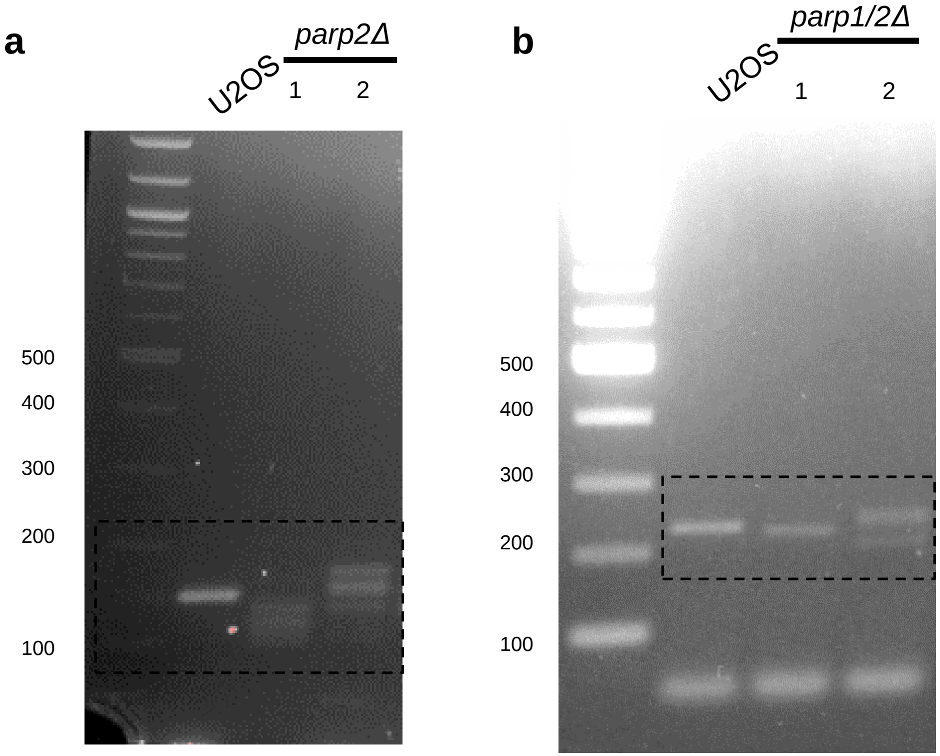

Supplementary Figure 13: Uncropped images of blots and gels

Supplementary Figure 4

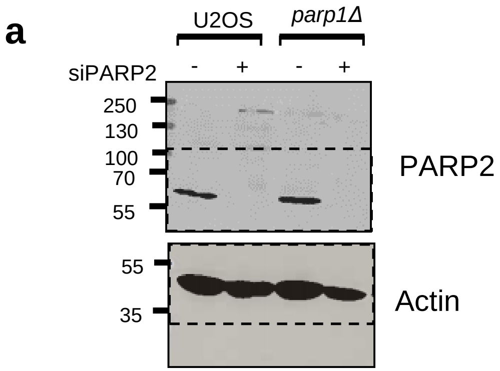

Supplementary Figure 5

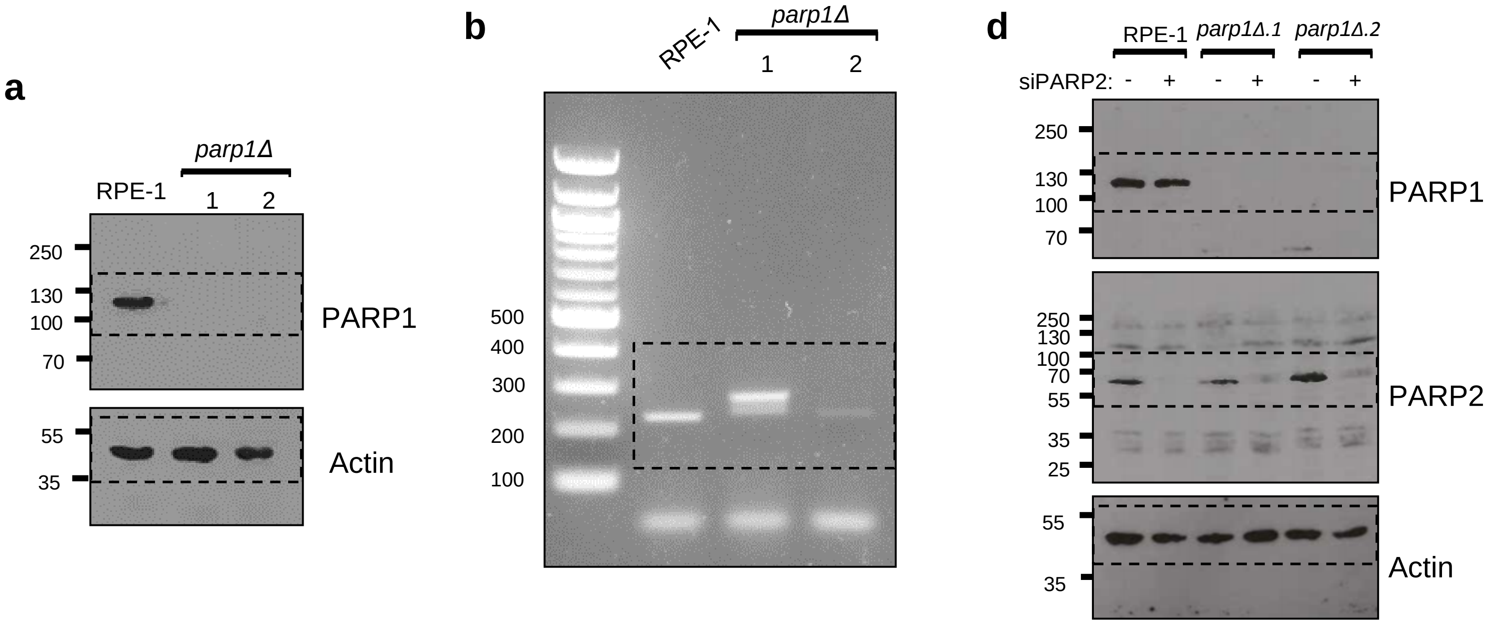

Supplementary Figure 13: Uncropped images of blots and gels

Supplementary Figure 7

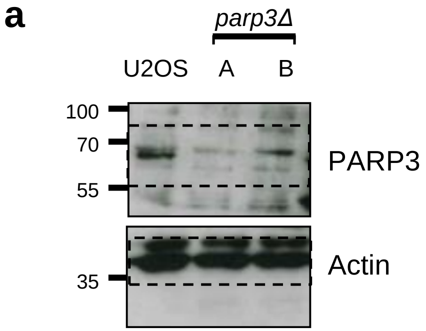

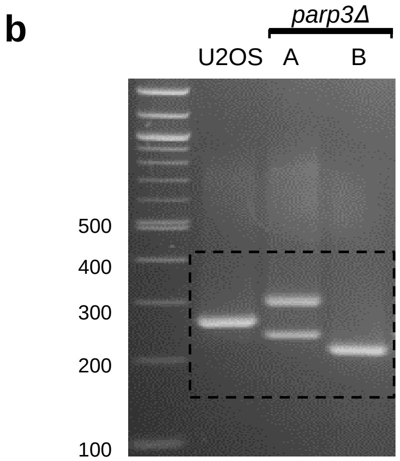

Supplementary Figure 8

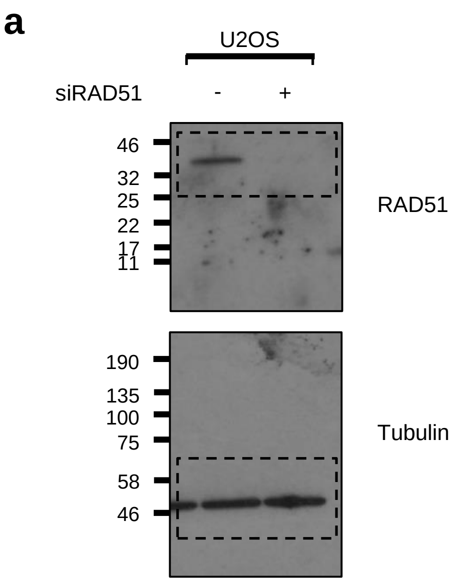

Supplementary Figure 9

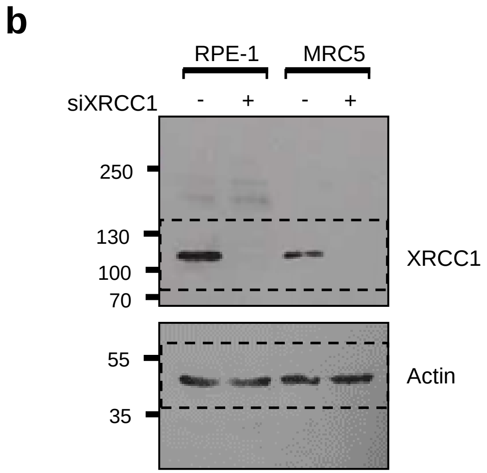

Supplementary Figure 13: Uncropped images of blots and gels
